# The role of total and cartilage-specific estrogen receptor alpha expression for the ameliorating effect of estrogen treatment on arthritis

**DOI:** 10.1186/ar4612

**Published:** 2014-07-15

**Authors:** Cecilia Engdahl, Anna E Börjesson, Huamei F Forsman, Annica Andersson, Alexandra Stubelius, Andree Krust, Pierre Chambon, Ulrika Islander, Claes Ohlsson, Hans Carlsten, Marie K Lagerquist

**Affiliations:** 1 Department of Rheumatology and Inflammation Research, Centre for Bone and Arthritis Research, Institute of Medicine, University of Gothenburg, Box 480, 405 30 Gothenburg, Sweden; 2 Department of Internal Medicine and Clinical Nutrition, Centre for Bone and Arthritis Research, Institute of Medicine, University of Gothenburg, Vita Stråket 11, 413 45 Gothenburg, Sweden; 3 Department of Functional Genomics, IGBMC, Collège de France, B.P. 10142, 674 04 Illkirch, France

## Abstract

**Introduction:**

Estrogen (E2) delays onset and decreases severity of experimental arthritis. The aim of this study was to investigate the importance of total estrogen receptor alpha (ERα) expression and cartilage-specific ERα expression in genetically modified mice for the ameliorating effect of estrogen treatment in experimental arthritis.

**Methods:**

Mice with total (total ERα^-/-^) or cartilage-specific (Col2α1-ERα^-/-^) inactivation of ERα and wild-type (WT) littermates were ovariectomized, treated with E2 or placebo, and induced with antigen-induced arthritis (AIA). At termination, knees were collected for histology, synovial and splenic cells were investigated by using flow cytometry, and splenic cells were subjected to a T-cell proliferation assay.

**Results:**

E2 decreased synovitis and joint destruction in WT mice. Amelioration of arthritis was associated with decreased frequencies of inflammatory cells in synovial tissue and decreased splenic T-cell proliferation. E2 did not affect synovitis or joint destruction in total ERα^-/-^ mice. In Col2α1-ERα^-/-^ mice, E2 protected against joint destruction to a similar extent as in WT mice. In contrast, E2 did not significantly ameliorate synovitis in Col2α1-ERα^-/-^ mice.

**Conclusions:**

Treatment with E2 ameliorates both synovitis and joint destruction in ovariectomized mice with AIA via ERα. This decreased severity in arthritis is associated with decreased synovial inflammatory cell frequencies and reduced splenic T-cell proliferation. ERα expression in cartilage is not required for estrogenic amelioration of joint destruction. However, our data indicate that ERα expression in cartilage is involved in estrogenic effects on synovitis, suggesting different mechanisms for the amelioration of joint destruction and synovitis by E2.

## Introduction

Rheumatoid arthritis (RA) is an autoimmune disease characterized by a massive infiltration of mononuclear and polymorphonuclear cells into the joints and the development of inflammation that results in synovitis and destruction of articular cartilage and adjacent bone loss [1].

The mechanisms that give rise to RA are only partly understood, and several different immune cells, including lymphocytes, macrophages, and neutrophils, are involved. Furthermore, a number of inflammatory mediators are implicated in the establishment and progression of arthritis, including proinflammatory cytokines such as tumor necrosis factor alpha (TNFα) and interleukin (IL)-6 and IL-17 [2,3].

RA affects 0.5% to 1% of the population [4], and the female-to-male ratio is 3:1. Estrogen plays an important role in the pathogenesis of RA, and we and others have demonstrated, in different experimental arthritis models, that estrogen delays the onset and decreases the severity of the disease [5-8]. However, the role of estrogen in inflammatory diseases is complex [9,10], and findings are contradictory; some human studies have reported no effect of estrogen, whereas we and others demonstrate beneficial effects of estrogen on RA [11-14]. Thus, there is a need to further understand the role of estrogen in RA.

The physiological effects of estrogen are exerted mainly via the two classic nuclear estrogen receptors (ERs): ERα and ERβ. Using ER-specific agonists, we have recently shown that the ameliorating effect of estrogen on collagen-induced arthritis is mediated mainly via ERα [5]. ERα is expressed in tissues adjacent to the joint inflammation, including cartilage and bone [15,16], as well as in other peripheral tissues involved in the immune response, including thymus and spleen [17,18]. However, it is not known via which cell type estrogen mediates its ameliorating effect on arthritis.

Since long-term estrogen treatment to postmenopausal women is associated with severe side effects, knowledge about the mechanism behind the protective effects of estrogen is of considerable importance in the search for new treatment strategies for arthritis. In the present study, antigen-induced arthritis (AIA) was used to examine the influence of estrogen on arthritis. AIA shares many of the histological features of RA, such as leukocyte infiltration and synovitis together with bone and cartilage destruction [19], and is dependent on helper T cells, synovial macrophages, and neutrophils but independent of cytotoxic T cells and B cells [20-23].

To determine the importance of ERα for arthritis, we induced AIA in genetically modified mice lacking ERα expression in all cells. Furthermore, since estrogen can exert direct effects on cartilage via ERα [16], we used mice lacking ERα specifically in chondrocytes to determine the importance of local cartilage-specific ERα expression for the protective effects of estrogen on arthritis. By evaluating the estrogenic response in these two models, we determined the importance of (a) total ERα expression and (b) cartilage-specific ERα expression in arthritis amelioration.

We demonstrate that estrogen ameliorates AIA via ERα. Furthermore, ERα expression in cartilage is not required for the amelioration of joint destruction. However, our data indicate that ERα expression in cartilage is involved in the ameliorating effects of estrogen on synovial inflammation.

## Materials and methods

### Animals

The ethics committee for animal experiments at the University of Gothenburg approved this study. Female mice were kept, 5 to 10 animals per cage, under standard environmental conditions and were fed with standard laboratory chow and tap water *ad libitum*.

In experiment 1, female C57BL/6 wild-type (WT) (Scanbur NOVA-SCB AB, Sollentuna, Sweden) mice were used. In experiment 2, total ERα inactivated mice (total ERα^-/-^) and WT littermates were used. Total ERα^-/-^ mice have a deletion in exon 3 of the ERα gene and do not express any of the isoforms of the ERα protein. The total ERα^-/-^ mice and corresponding WT (ERα^+/+^) littermates were inbred C57BL/6 mice and generated by breeding male ERα^+/-^ with female ERα^+/-^ mice, obtained as previously described [24]. In experiment 3, cartilage-specific ERα inactivated mice and WT littermates were used. The generation of cartilage-specific ERα inactivated mice has previously been described, and these mice have a 62% reduction in ERα protein levels in cartilage but no reduction in bone or liver [25]. Briefly, mice in which exon 3 of the ERα gene is flanked by loxP sequences (ERα^flox/flox^) were crossed with Col2α1-Cre mice [26] to generate Col2α1-Cre;ERα^flox/+^ mice. These mice were crossed with ERα^flox/flox^ mice to generate conditional mutants (Col2α1-Cre;ERα^flox/flox^, hereafter referred to as Col2α1-ERα^-/-^) and corresponding littermate controls (ERα^flox/flox^). The ERα^flox/flox^ mice are inbred C57BL/6 mice, and the Col2α1-Cre mice were generated on a B6SJLF1 background and backcrossed more than six times with C57BL/6 mice.

### Ovariectomy and hormone treatment

Total ERα^-/-^ mice have increased sex steroid levels because of disturbed negative feedback regulation. To avoid confounding endogenous sex steroid effects and to imitate a postmenopausal state, all mice were ovariectomized and treated with placebo or estrogen. Ovariectomy (ovx) was performed at 10 weeks of age through a midline incision of the skin and flank incisions of the peritoneum. The skin incision was closed with metallic clips. Surgery was performed under anesthesia with isoflurane (Pfizer AB, Täby, Sweden). Carprofen (OrionPharma, Espoo, Finland) was used post-operatively as an analgesic.

Ovx mice were inserted with a subcutaneous slow-release pellet (Innovative Research of America, Sarasota, FL, USA) with 17β-estradiol (E2) (0.83 μg/day) or placebo. Mice treated with E2 in a dose similar to the one used in this study obtain serum E2 levels of approximately 60 pg/mL [27]. In mice, normal serum levels of E2 vary between 25 and 50 pg/mL in diestrus and between 150 and 200 pg/mL in estrus [28]. Thus, the dose used in this study resulted in physiological serum E2 levels. Treatment started at the time of ovx and lasted until termination.

### Induction of antigen-induced arthritis

The mice were immunized with 0.2 mg of methylated bovine serum albumin (mBSA) (Sigma-Aldrich, Stockholm, Sweden) dissolved in phosphate-buffered saline (PBS) and emulsified with an equal volume of complete Freund’s adjuvant (Sigma-Aldrich). A total volume of 100 μL was injected intradermally at the base of the tail (50 μL on each side). After 7 days, mice received an injection with 0.3 mg of mBSA dissolved in 30 μL of vehicle (20% dH_2_O and 80% saline) into one knee joint.

### Tissue collection and histological examination

Fourteen days after the primary immunization, the mice were anaesthetized with Ketalar^®^/Domitor (PfizerAB), bled, and killed by cervical dislocation. Serum was individually stored at -20°C until use. Uteri were collected and weighed. The knees were separately placed in 4% formaldehyde, decalcified, and embedded in paraffin. Sections were stained with eosin/hematoxylin, and a blinded examiner (CE) graded synovitis and joint destruction. Synovial hypertrophy was defined as a membrane thickness of more than two cell layers [29]. A histological scoring system was used as follows: mild (1), moderate (2), and severe (3) synovitis and joint destruction. Bone erosions and cartilage degradation were considered separately in joint destruction.

### Single-cell preparation and cellularity measurement

Spleens were harvested, and single-cell suspensions were obtained after the tissue was mashed and passed through a 70-μm cell strainer (Becton Dickinson Biosciences Pharmingen, San Diego, CA, USA) in 15 mL of PBS. Pelleted cells from spleen were resuspended in Tris-buffered 0.83% NH_4_Cl solution to lyse erythrocytes and washed in PBS. The total number of spleen leukocytes was analyzed in complete medium (Iscoves medium with L-glutamin, mercaptoethanol, gentamycin, and fetal calf serum) (Sigma-Aldrich) by using an automated cell counter (Sysmex, Hamburg, Germany). Synovial tissue was dissected and placed in medium (RPMI) (FisherScientific, Västra Frölunda, Sweden). Medium with DNaseI (Sigma-Aldrich) and collagenase type IV (Roche AB, Stockholm, Sweden) was added, and the suspension was incubated for 1 hour at 37°C. A single-cell suspension was obtained after the tissue was mashed and passed through a 40-μm cell strainer (Becton Dickinson) in 4 mL of PBS.

### Concanavalin A-induced T-cell proliferation in spleen cell cultures

Isolated splenocytes from experiments 1 to 3 were suspended in complete medium (1 × 10^6^ cells per mL) and cultured in round-bottom plates (Nunc, Roskilde, Denmark) at 37°C, 5% CO_2_, and 95% humidity. The T-cell mitogen concanavalin A (conA) (Sigma-Aldrich) was added to the medium in a final concentration of 1.25 μg/mL, and cells with no added mitogen were used as controls. After 48 hours of culture, supernatants were taken from the wells. After an additional 2 hours (in total, 50 hours), 1 μCi[3H] thymidine (Amersham Pharmacia Biotech, Uppsala, Sweden) was added for 12 hours. Cells were harvested onto glass fiber filters and counted in a β-counter (PerkinElmer, Waltham, MA, USA). Cell cultures were set in triplicates, and results are presented as proliferation index (median of count per minute in wells with conA minus the median of count per minute in control wells).

### Cytokine analysis

Cytokines from the conA-stimulated and control spleen cell culture supernatants were analyzed by using a Mouse Th1/Th2 10 plex FlowCytomix Multiplex kit (eBioscience, San Diego, CA, USA). The cytokine levels of control supernatants were subtracted from the conA-stimulated cytokine levels.

### Flow cytometry for analysis of cell phenotype

Isolated splenocytes were stained for analysis by using the following antibodies: BD Horizon V450-conjugated anti-CD4 (Becton Dickinson) and fluorescein isothiocyanate (FITC)-conjugated anti-CD8 (Becton Dickinson). Synovial cells were examined by using BD Horizon V450-conjugated anti-CD11b (Becton Dickinson), FITC-conjugated anti-CD3 (Becton Dickinson), allophycocyanin (APC)-conjugated anti-F4/80 (BioLegend, San Diego, CA, USA), Peridinin Chlorophyll Protein Complex (PerCP)-conjugated anti-CD19 (BioLegend), and phycoerythrin (PE)-conjugated anti-Gr1. Labeled cells were analyzed in a FACS CantoII (Becton Dickinson). FlowJo version 8.5.2 (Tree Star, Ashland, OR, USA) software was used for analyzing the data.

### Measurement of mBSA antibodies

Serum was analyzed for mBSA antibodies. IgG type Low bind Microplates (Nunc) were precoated with 0.1 g/mL mBSA at 4°C overnight. After coating, the plates were washed, blocked with 2% casein, and incubated with the serum. Titration experiments were performed in order to find the optimal dilution of the sera for enzyme-linked immunosorbent assay (ELISA) analysis. Biotinylated F(ab′)2 fragments of goat anti-mouse IgG (Jackson ImmunoResearch Laboratories, West Grove, PA, USA) were used as secondary antibody. Bound IgG was detected with a peroxidase-conjugated anti-mouse IgG (ICN Biochemicals, Aurora, OH, USA) and tetramethylbenzidine (Sigma-Aldrich) substrate. Absorbance was measured at 450 nm on a SpectraMax 340 DC spectrophotometer (Molecular Devices, Sunnyvale, CA, USA).

### Real-time polymerase chain reaction

RNA was isolated from the epiphyseal part of the femur, containing mainly articular and growth plate chondrocytes and trabecular bone, by using an RNeasy kit (Qiagen, Chatswort, CA, USA). Amplifications were performed by using the Applied Biosystem StepOnePlus™ Real-Time PCR System (PE, Applied Biosystems, Stockholm, Sweden) and Assay-on-Demand primer and probe sets (PE, Applied Biosystems), labeled with reporter fluorescent dye FAM. Predesigned primers and probe labeled with reporter fluorescent dye VIC, specific for 18S ribosomal RNA, were included in the reaction as an internal standard. The assay identification numbers were chemokine CXC motif ligand 1 (CXCL1): Mm04207460_m1 and chemokine CXC motif ligand 5 (CXCL5): Mm00436451_g1. The amount of mRNA was calculated by using a standard curve method in accordance with the instructions of the manufacturer (PE, Applied Biosystem) and adjusted for 18S ribosomal RNA.

### Preparation of peritoneal exudate polymorphonuclear leukocytes

Inflammation was induced by injecting 0.1 mL of uric acid solution (10% uric acid in saline) into the peritoneal cavity of intact Col2α1-ERα^-/-^ and WT littermates [30]. Before injection, the uric acid solution was sonicated for 10 minutes and shaken vigorously. Peritoneal exudate cells were harvested 4 hours after injection by lavages with cold PBS. Hypotonic lysis was performed after centrifugation (200 *g* for 10 minutes at 4°C) to eliminate red blood cells, and the cells were washed in Krebs-Ringer glucose buffer. Nuclear staining using a Scepter™ (EMD Millipore, Billerica, MA, USA) determined the numbers of peritoneal exudate cells. The purity of the uric acid-induced peritoneal exudate cells was 95% polymorphonuclear, and the remaining cell population was composed of mononuclear cells [30].

### Statistical analysis

For statistical evaluation, Student *t* tests were performed for comparison of two independent groups. The histological scoring was performed by using an ordinal scale system requiring non-parametric statistical evaluation and therefore the Mann-Whitney *U* test was used.

## Results

### Estrogen decreases antigen-induced arthritis severity

**Figure 1:**
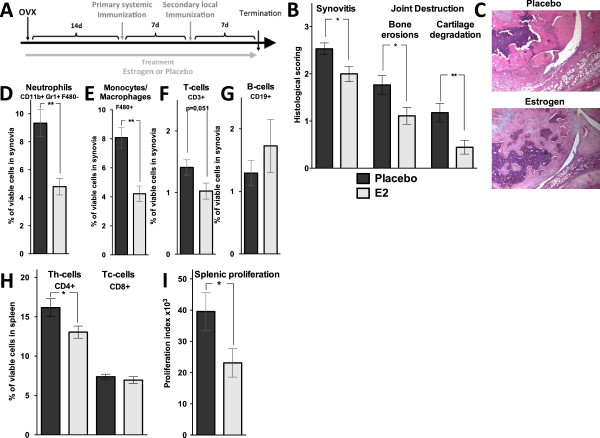
**Estrogen ameliorates antigen-induced arthritis (AIA). (A)** Scheme for AIA. Total ERα^-/-^, Col2α1-ERα^-/-^, and wild-type (WT) mice were ovariectomized (OVX) at 10 weeks of age, followed by insertion of slow-release treatment pellets with placebo or estrogen (E2) (0.83 μg/day). Fourteen days after ovariectomy, the animals were immunized by an intradermal injection of methylated bovine serum (mBSA) emulsified in Freund’s complete adjuvant (0.2 mg mBSA) and 7 days later given an intra-articular (IA) injection of mBSA (0.3 mg mBSA dissolved in 30 μL of vehicle) in one knee joint. Seven days after the IA injection, the mice were bled and terminated. **(B)** Knee joints obtained at termination of WT mice with AIA treated with placebo or estrogen were examined with a histological scoring of synovitis and joint destruction. The histological examination was performed by using a scale from 1 to 3: 1, mild; 2, moderate; and 3, severe. *P <0.05, **P <0.01, Mann-Whitney *U* test. (n = 17-18). **(C)** Representative images of joint histology. Percentage of viable **(D)** neutrophils (CD11b^+^, Gr1^+^, F480^-^), **(E)** monocytes/macrophages (F480^+^), **(F)** T cells (CD3^+^), and **(G)** B cells (CD19^+^) in synovia tissues. *P <0.05, Student *t* test (n = 7-8). **(H)** Percentage of viable CD4^+^ Th (T helper) cells and CD8^+^ Tc (T cytotoxic) cells in the spleen. *P <0.05, Student *t* test. (n = 17-18). **(I)** Proliferation of splenocytes after stimulation with the T-cell mitogen concanavalin A (conA). Cell cultures were set in triplicates, and results are presented as proliferation index (median of count per minute in wells with conA minus the median of count per minute in control wells). *P <0.05, Student *t* test (n = 17-18). Results are shown as mean ± standard error of the mean.

Placebo- and E2-treated ovx mice (experiment 1, see Materials and methods) were challenged with antigen according to the regimen illustrated in Figure 1A. E2 treatment was confirmed by increased uterus weight compared with placebo (740%, *P* <0.001). Placebo-treated ovx mice developed severe arthritis in the immunized knee joint, as revealed by massive cellular infiltration into the joint space, synovial inflammation, and joint destruction (Figure 1B,C). Histological examination of joints from E2-treated ovx mice showed significantly less joint destruction (bone erosions: -37%, *P* <0.05; cartilage degradation: -62%, *P* <0.01) and synovial inflammation (-21%, *P* <0.05) compared with placebo-treated mice, demonstrating that E2 treatment ameliorates disease severity in ovariectomized mice (Figure 1B,C).

Synovial cell populations were investigated by using flow cytometry. E2 treatment significantly decreased the frequency of neutrophils (-49%, *P* <0.01) and monocytes/macrophages (-58%, *P* <0.01) compared with placebo treatment (Figure 1D,E). No significant effects were detected in lymphocyte frequencies (Figure 1F,G), although a tendency to decreased T-cell frequency (-28%, *P* = 0.051) was found (Figure 1F). Furthermore, the antigen-specific antibody response was investigated by measuring serum levels of mBSA-specific IgG, and similar levels were found in the placebo- and E2-treated animals (data not shown). These results suggest that neutrophils and monocytes/macrophages are involved on a local level in the ameliorating effect of E2 on AIA.

Splenocytes from AIA-induced animals were investigated by using flow cytometry to determine the frequencies of CD4^+^ helper T (Th) cells and CD8^+^ cytotoxic T (Tc) cells. There was a significant reduction in the frequency of Th cells (-25%, *P* <0.05), but no effect on Tc cell frequency was seen after E2 treatment (Figure 1H). These data are in accordance with the fact that AIA is dependent on Th, and not Tc, cells. Splenic proliferation was assessed after activation with the T-cell mitogen conA. E2 treatment decreased the proliferation (-32%, *P* <0.05) compared with placebo treatment (Figure 1I). To further investigate the effect of E2 on the peripheral immune response, the cytokine profile was investigated in supernatants of conA-stimulated spleen cell cultures. The supernatants from E2-treated animals showed reduced levels of IL-17 (-51%, *P* <0.01), IL-2 (-27%, *P* <0.05), interferon-gamma (IFN-γ) (-23%, *P* <0.05), and IL-6 (-28%, *P* <0.05) versus placebo treatment, whereas levels of IL-4, TNFα, and GM-CSF (granulocyte macrophage colony-stimulating factor) were unchanged (Table 1), demonstrating that E2 treatment results in an altered systemic cytokine profile which may be important in the amelioration of arthritis.

**Table 1:** Cytokines in supernatants from spleen cell proliferation cultures

		**(Exp. 1)**	**(Exp. 2) Total ERα**^**-/-**^		**(Exp. 3) Col2α1-ERα**^**-/-**^	
**(pg/mL)**		**WT**	**WT**	**KO**	**WT**	**KO**
**IL-17**	**P**	850 ± 107	158 ± 33	97 ± 23	785 ± 158	715 ± 124
	**E2**	417 ± 80^a^	46 ± 18^a^	130 ± 41	391 ± 79^b^	335 ± 103^b^
**IFNγ**	**P**	1,039 ± 69	8,881 ± 2,865	7,294 ± 3,192	4,465 ± 1,070	4,782 ± 760
	**E2**	800 ± 54^b^	2,813 ± 528^b^	4,926 ± 694	1,622 ± 360^b^	2,697 ± 340^b^
**IL-2**	**P**	1,038 ± 83	4,355 ± 581	4,098 ± 540	7,128 ± 1,598	4,405 ± 1,085
	**E2**	759 ± 64^b^	2,697 ± 663^c^	3,120 ± 640	1,247 ± 403^b^	531 ± 134^b^
**IL-6**	**P**	644 ± 48	596 ± 85	475 ± 127	523 ± 81	425 ± 59
	**E2**	469 ± 59^b^	383 ± 66^d^	447 ± 57	326 ± 68	227 ± 56^b^
**IL-4**	**P**	337 ± 80	114 ± 12	95 ± 9	120 ± 38	122 ± 48
	**E2**	279 ± 41	90 ± 9	102 ± 12	141 ± 30	102 ± 62
**TNFα**	**P**	147 ± 27	95 ± 18	105 ± 15	169 ± 32	125 ± 15
	**E2**	190 ± 73	109 ± 31	94 ± 15	122 ± 20	88 ± 22
**GM-CSF**	**P**	195 ± 22	877 ± 268	1,011 ± 202	430 ± 162	719 ± 361
	**E2**	158 ± 123	1,118 ± 246	826 ± 152	472 ± 189	1,069 ± 229

The cytokine profile was investigated in supernatants of concanavalin A-stimulated spleen cell cultures from experiments 1 (n = 9-10), 2 (n = 12-17), and 3 (n = 8-11) (see Materials and methods). Values are given as mean ± standard error of the mean. ^a^*P* <0.01, ^b^*P* <0.05 versus placebo, Student *t* test, ^c^*P* = 0.075, ^d^*P* = 0.06. E2, estrogen; Exp., experiment; IFNγ, interferon gamma; IL, interleukin; KO, knockout; P, placebo; GM-CSF, granulocyte macrophage colony-stimulating factor; TNFα, tumor necrosis factor alpha; WT, wild-type.

### Estrogen ameliorates antigen-induced arthritis via ERα

**Figure 2:**
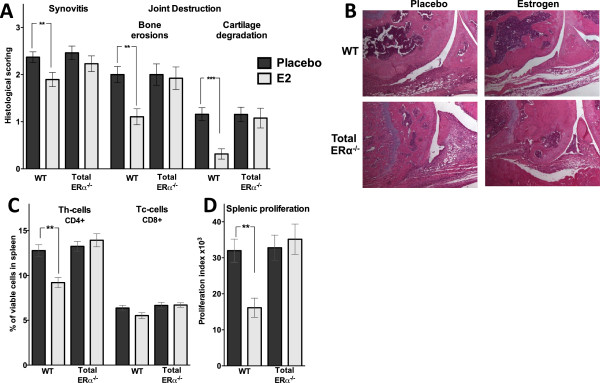
**Estrogen ameliorates antigen-induced arthritis (AIA) via estrogen receptor alpha (ERα).** Total ERα^-/-^ and wild-type (WT) littermates were treated with placebo or estrogen (E2) (0.83 μg/day) and induced with AIA. **(A)** Histological scoring of synovitis and joint destruction. The histological examination was performed by using a scale from 1 to 3: 1, mild; 2, moderate; and 3, severe. *P <0.05, **P <0.01, ***P <0.001 Mann-Whitney *U* test. **(B)** Representative images of joint histology. **(C)** Percentage of viable CD4^+^ Th (T helper) cells and CD8^+^ Tc (T cytotoxic) cells in the spleen. **P <0.01, Student *t* test. **(D)***In vitro* proliferation of splenocytes, harvested from mice with AIA treated with placebo or E2 (0.83 μg/day), after stimulation with the T-cell mitogen concanavalin A (conA). Cell cultures were set in triplicates, and results are presented as proliferation index (median of count per minute in wells with conA minus the median of count per minute in control wells). *P <0.05, Student *t* test. Results are shown as mean ± standard error of the mean (n = 14-19).

We have previously shown that ERα is important for the ameliorating effects of estrogen in collagen-induced arthritis and immune-mediated bone loss, using estrogen receptor-specific agonists [5]. To confirm the requirement of ERα for the ameliorating effects of E2 treatment on arthritis by using a genetically modified animal model, total ERα^-/-^ and their corresponding WT littermates (experiment 2, see Materials and methods) were challenged with antigen according to the regimen illustrated in Figure 1A. E2 treatment significantly increased the uterus weight of WT littermates (805%, *P* <0.001), but not total ERα^-/-^ mice, compared with placebo. There were no significant differences between placebo-treated WT and total ERα^-/-^ mice regarding synovitis or joint destruction, demonstrating that unliganded ERα is not involved in the development of AIA. As expected, histological examination of placebo-treated joints in WT littermates revealed severe arthritis in the immunized knee joints and E2 treatment reduced joint destruction (bone erosions: -45%, *P* <0.01; cartilage degradation: -73%, *P* <0.001) as well as synovial inflammation (-20%, *P* <0.01) (Figure 2A,B). In contrast, E2 treatment did not ameliorate synovitis or joint destruction in total ERα^-/-^ animals (Figure 2A,B). This finding demonstrates that ERα is required for the ameliorating effect of E2 in AIA.

Splenocytes from total ERα^-/-^ mice and WT littermates were investigated by using flow cytometry. E2 reduced the frequency of Th cells, but not Tc cells, in WT mice (-28%, *P* <0.01), whereas no estrogenic effect was detected in total ERα^-/-^ animals (Figure 2C). Proliferation was assessed in splenocytes after activation with the T-cell mitogen conA. As expected, E2 reduced the proliferation (-50%, *P* <0.01) compared with placebo treatment in WT littermates, whereas no estrogenic effect was detected in total ERα^-/-^ animals compared with placebo (Figure 2D). The cytokine profile in supernatants of the proliferation cultures showed significantly reduced levels of IL-17 (-71%, *P* <0.01) and IFN-γ (-68%, *P* <0.05) and tendencies to reduced levels of IL-2 (-38%, *P* = 0.075) and IL-6 (-36%, *P* = 0.06) in WT littermates after E2 treatment compared with placebo treatment (Table 1). In contrast, E2 did not influence the cytokine pattern in total ERα^-/-^ animals. These results demonstrate that ERα is required for the reduced frequency of systemic Th cells, the reduced T-cell proliferation, and the altered cytokine profile seen after E2 treatment.

### ERα in cartilage is not required for the ameliorating effects of estrogen on joint destruction but is involved in the ameliorating effects of estrogen on synovial inflammation

**Figure 3:**
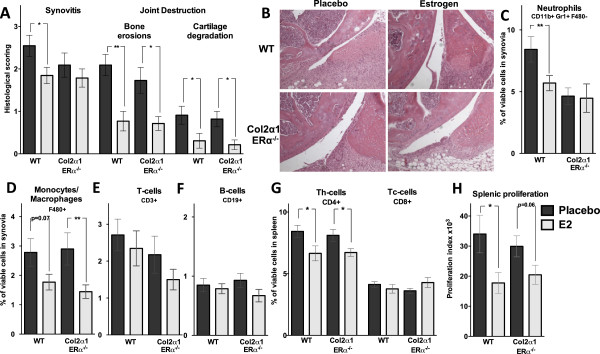
**Estrogen receptor alpha (ERα) in cartilage is involved in the estrogenic amelioration of synovitis but not joint destruction.** Histological examination was performed on knee joints from Col2α1-ERα^-/-^ and wild-type (WT) littermates with antigen-induced arthritis (AIA), treated with estrogen (E2) (0.83 μg/day) or placebo, to determine effects on inflammation and joint destruction. **(A)** Histological scoring of synovitis and joint destruction. The histological examination was performed by using a scale from 1 to 3: 1, mild; 2, moderate; and 3, severe. *P <0.05, **P <0.01, Mann-Whitney *U* test (n = 11-14). **(B)** Representative images of joint histology. Percentage of viable **(C)** neutrophils (CD11b^+^, Gr1^+^, F480^-^), **(D)** monocytes/macrophages (F480^+^), **(E)** T cells (CD3^+^), and **(F)** B cells (CD19^+^) in synovia tissues. *P <0.05, Student *t* test (n = 6-10). **(G)** Percentage of viable CD4^+^ Th (T helper) cells and CD8^+^ Tc (T cytotoxic) cells in the spleen. *P <0.05, Student *t* test (n = 11-14). **(H)***In vitro* proliferation of splenocytes, harvested from mice with AIA treated with placebo or E2 (0.83 μg/day), after stimulation with the T-cell mitogen concanavalin A (conA). Cell cultures were set in triplicates, and results are presented as proliferation index (median of count per minute in wells with conA minus the median of count per minute in control wells). *P <0.05, Student *t* test. Results are shown as mean ± standard error of the mean (n = 11-14).

Chondrocytes express ERα, and estrogen can exert direct effects on these cells [16]. Expression of ERα in cartilage therefore may be involved in the ameliorating effect of estrogens on AIA. Col2α1-ERα^-/-^ mice, lacking ERα expression specifically in chondrocytes, and their corresponding WT littermates (experiment 3, see Materials and methods) were challenged with antigen according to the regimen illustrated in Figure 1A. Treatment with E2 significantly induced the uteri weight compared with placebo in both WT (938%, *P* <0.001) and Col2α1-ERα^-/-^ (724%, *P* <0.001) mice. As seen for total ERα^-/-^ animals, there were no significant differences between placebo-treated WT and Col2α1-ERα^-/-^ mice regarding synovitis or joint destruction, demonstrating that unliganded ERα in chondrocytes is not involved in the development of AIA. E2 treatment of both WT and Col2α1-ERα^-/-^ mice decreased bone erosions (-63% and -59%, respectively; *P* <0.01) and cartilage degradation (-66% and -74%, respectively; *P* <0.05) (Figure 3A,B). Interestingly, E2 decreased synovitis in WT mice (-28%, *P* <0.05) but had no significant effect on synovitis in Col2α1-ERα^-/-^ animals (Figure 3A,B). These results suggest that ERα expression in cartilage is involved in the protective effects of E2 on synovial inflammation but that ERα in cartilage is dispensable for the effect of E2 on joint destruction. These results were repeated in an additional experiment (n = 11-13) with similar results (data not shown).

Flow cytometry analysis of splenocytes demonstrated reduced Th cell frequency after E2 treatment in both WT (-21%, *P* <0.05) and Col2α1-ERα^-/-^ (-17%, *P* <0.05) mice, whereas Tc cells were not affected by E2 treatment (Figure 3G). E2 decreased the proliferative response in splenocytes after conA stimulation in WT littermates (-45%, *P* <0.05) compared with placebo, and there was a tendency of decreased proliferative response also in the Col2α1-ERα^-/-^ mice (-33%, *P* = 0.06) (Figure 3H). Furthermore, E2 affected the cytokine profile in supernatants of the spleen cell proliferation cultures similarly in WT and Col2α1-ERα^-/-^ mice with reduced levels of IL-2, IL-17, and IFN-γ (Table 1). Thus, ERα expression in cartilage does not influence the systemic immune response to E2 in AIA.

**Figure 4:**
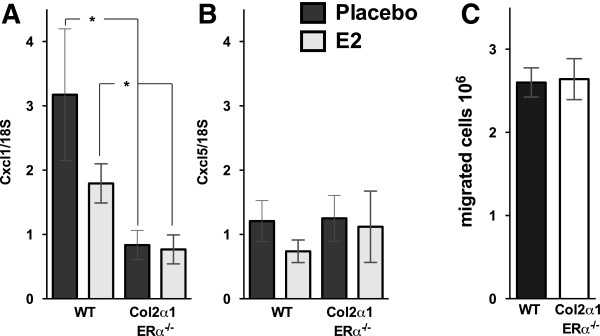
**Estrogen receptor alpha (ERα) expression in cartilage affects local gene expression of the neutrophil chemokine CXCL1 but has no effect on systemic neutrophil migration capacity.** Col2α1-ERα^-/-^ and wild-type (WT) littermates were treated with placebo or estrogen (E2) (0.83 μg/day) and induced with antigen-induced arthritis. Expression of **(A)** Cxcl1 and **(B)** Cxcl5 in the epiphyseal part of the femur. The mRNA level of each gene was adjusted for expression of 18S rRNA. **(C)** Neutrophil migration was evaluated by analysis of the number of peritoneal exudate cells after induction of inflammation in the peritoneal cavity by using uric acid. *P <0.05, Student *t* test. Results are shown as mean ± standard error of the mean (n = 4-8).

Synovial cells were investigated by using flow cytometry, and E2 treatment reduced the monocyte/macrophage frequency in Col2α1-ERα^-/-^ mice compared with placebo (-50%, *P* <0.05), and there was a tendency to decrease in WT mice compared with placebo (-36%, *P* = 0.07) (Figure 3D). No effects were detected in lymphocyte frequencies (Figure 3E,F). However, E2 treatment decreased the neutrophil frequency in WT mice compared with placebo (-32%, *P* <0.05), whereas no E2 effect on neutrophils was detected in Col2α1-ERα^-/-^ mice compared with placebo (Figure 3C). A comparison between the placebo-treated mice showed that the frequency of neutrophils was significantly decreased in Col2α1-ERα^-/-^ mice compared with WT littermates (-45%, *P* <0.05). To investigate the cause of the decreased neutrophil frequency in the synovia in Col2α1-ERα^-/-^ mice, we analyzed the expression of neutrophil chemoattractants. Expression of the neutrophil chemoattractants CXCL1 and CXCL5 was investigated in the epiphyseal part of the femur, close to the arthritic joint. E2 treatment did not affect CXCL1 or CXCL5 expression in either WT or Col2α1-ERα^-/-^ mice (Figure 4A,B). However, the CXCL1 expression was significantly decreased in Col2α1-ERα^-/-^ mice compared with WT littermates both after placebo treatment (-72%, *P* <0.05) and after E2 treatment (-57%, *P* <0.05) (Figure 4A).

The systemic neutrophil migration capability was investigated in Col2α1-ERα^-/-^ mice and WT littermates by inducing peritoneal inflammation and investigating the number of neutrophils in the peritoneal exudate. The neutrophil count was equal between WT and Col2α1-ERα^-/-^ mice (Figure 4C), suggesting that there is no difference in the systemic neutrophil responsiveness in mice lacking ERα expression specifically in chondrocytes compared with WT littermates.

## Discussion

RA is an autoimmune disease characterized by chronic inflammation, which leads to destruction of cartilage and bone loss. Several studies have shown that estrogen exerts positive effects on arthritis and arthritis-related immune-mediated bone loss [13,14], but owing to negative side effects, estrogen is not considered suitable as a long-term treatment. It is therefore of importance to determine the mechanism behind its protective effects on arthritis in order to develop new treatment strategies which retain the positive, but not the negative, effects of estrogen. We demonstrate that estrogen (E2) treatment ameliorates both synovitis and joint destruction in AIA via ERα and that ERα expression in cartilage cells is dispensable for the estrogenic protection against joint destruction. Interestingly, our data indicate that ERα expression in cartilage is involved in the estrogenic effects on synovitis.

Estrogen signaling is complex, involving two classic nuclear receptors: ERα and ERβ. We and others have previously reported that activation of ERα, using selective ERα agonists, decreases the severity of both collagen-induced arthritis and AIA [5,31,32]. In the present study, we have used genetically modified mouse models, which are on a genetic background rendering them relatively insensitive to collagen-induced arthritis, and therefore we have used AIA as a model of arthritis in order to effectively assess effects on arthritis severity. We show that total deletion of ERα expression inhibits the protective effects of E2 on both synovitis and joint destruction, demonstrating the crucial role of ERα for the ameliorating effect of estrogen on arthritis in female mice. ERβ activation has also been implicated to play a role in the amelioration of arthritis in males [32,33]. However, we and others have shown, in both female and male rodents, that ERα, and not ERβ, activation ameliorates arthritis [5,31]. Furthermore, in the present study, E2 treatment was not able to ameliorate arthritis in ERα inactivated mice despite the presence of ERβ, demonstrating that ERα is the main receptor mediating the protective effects of estrogen on arthritis.

T cells are of particular importance in AIA and this is supported by the fact that nude mice lacking T cells cannot be induced with AIA [22]. Estrogen affects T lymphopoiesis, and we and others have previously demonstrated that estrogenic repression of T lymphopoiesis is mediated via ERα [27,34]. Estrogen has also been shown to decrease systemic T lymphocyte proliferation [35], and herein we confirm these results and establish that expression of ERα is required for the decreased conA-stimulated splenic T-cell proliferation. In this study, we demonstrate that E2 decreases the frequency of splenic Th cells via ERα, whereas Tc cells are unaffected. These results are in accordance with the notion that AIA is a Th-dependent disease [22]. Our study shows a clear estrogenic effect on systemic Th cells found in the spleen, whereas the local T cells, in the inflamed synovial membrane, are not significantly affected. It has previously been reported that systemic, rather than local, counteraction of Th cell function is responsible for the beneficial effects of Th cell depletion in the AIA model [36], and we suggest that systemic, rather than local, effects on Th cells are involved in the therapeutic efficiency of E2 in AIA. Evaluation of the cytokines produced by splenocytes treated with the T-cell mitogen conA demonstrated that E2 treatment reduces the levels of IFN-γ, IL-2, IL-6, and IL-17 and that this reduction is mediated via ERα. These cytokines are all implicated in the pathogenesis of arthritis [37,38] and may be involved in the ameliorating effect of estrogen on arthritis. AIA is not dependent on B cells, except in very severe cases [23]. Accordingly, we found that neither antigen-specific antibodies nor the B-cell frequency in synovia was associated with the estrogenic amelioration of arthritis.

The role of chondrocytes in the pathogenesis of arthritis has gained increasing interest. The recent finding that synovial fluid from patients with RA triggers secretion of various cytokines from chondrocytes, including cytokines involved in chemotactic migration of immune cells, suggests that these cells can actively take part in inflammatory joint diseases and not just be passive participators, as previously believed [39,40]. Chondrocytes express ERs, and estrogen can affect protein production in these cells [16]. We have recently shown that ERα expression in cartilage is important for the reduction of growth plate height induced by high E2 levels in adult mice, using a cartilage-specific ERα inactivated mouse model (Col2α1-ERα^-/-^ mice) [25]. In this study, we used the Col2α1-ERα^-/-^ mice to determine the importance of cartilage-specific ERα expression for the ameliorating effects of estrogen on arthritis. E2 treatment of Col2α1-ERα^-/-^ mice resulted in a similar reduction in joint destruction as seen in WT littermates, demonstrating that ERα expression in chondrocytes is not involved in the estrogenic protection against joint destruction. Interestingly, E2 treatment did not significantly reduce the synovial inflammation in mice lacking ERα expression in chondrocytes, suggesting different target cells and mechanisms for the estrogenic protection against synovial inflammation and joint destruction. Dissociation between synovial inflammation and joint destruction has been described before where treatments have provided protection against bone erosions despite continuous synovial inflammation [41], and progression of bone erosions has been detected despite improvement of inflammatory scores [42]. In an attempt to understand the mechanism behind the lack of estrogenic response on synovial inflammation in Col2α1-ERα^-/-^ mice, we examined synovial cell populations. Synovial monocytes/macrophages and neutrophils are known to be important in RA and produce cytokines and chemokines that attract and further activate the immune system [43,44], and estrogen regulates both the number and function of neutrophils as well as reduces neutrophil chemotaxis [45]. We show that E2 treatment reduced the frequency of monocytes/macrophages in the joints both in WT littermates and in Col2α1-ERα^-/-^ mice. In contrast, E2 treatment was only able to decrease the frequency of neutrophils in the joints of WT littermates, whereas the neutrophils were unaffected by E2 treatment in mice lacking ERα expression in chondrocytes. Thus, the inability of E2 to significantly decrease synovitis in mice lacking ERα expression in cartilage cells may depend on the lack of E2 effect on neutrophils. The lack of E2 effect on neutrophils might be influenced by the fact that the number of neutrophils in the placebo-treated cartilage-specific ERα inactivated mice compared with the placebo-treated WT mice was significantly lower, suggesting that ERα expression in cartilage might be involved in the migration of neutrophils. Accordingly, we found that expression of the neutrophil chemokine CXCL1 [46,47], known to be expressed in chondrocytes [48,49], was decreased in the Col2α1-ERα^-/-^ mice, whereas no significant difference was detected in the expression of the neutrophil chemokine CXCL5, known to be expressed in cells of the myeloid lineage, including monocytes and osteoclasts [50]. Our data indicate that ERα expression in chondrocytes is required for normal expression of CXCL1 and thus for normal local migration of neutrophils into the joint. In addition, we found no differences in the systemic neutrophil responsiveness in the Col2α1-ERα^-/-^ mice, which indicates that only the local migration of neutrophils into the arthritic joint is altered in the Col2α1-ERα^-/-^ mice.

## Conclusions

ERα is required for the protective effects of estrogen treatment of AIA in ovx animals. Furthermore, ERα expression in cartilage is not required for the estrogenic amelioration of joint destruction. However, our data indicate that ERα expression in cartilage is involved in estrogenic effects on synovitis by influencing neutrophil migration, suggesting different mechanisms for the amelioration of joint destruction and synovitis by E2. Increased knowledge about the mechanisms behind the beneficial effects of estrogen is useful in the search for novel treatments against arthritis.

## Abbreviations

AIA: antigen-induced arthritis; conA: Concanavalin A; E2: estrogen; ER: estrogen receptor; FITC: fluorescein isothiocyanate; IFN-γ: interferon-gamma; IL: interleukin; mBSA: methylated bovine serum albumin; ovx: ovariectomy; PBS: phosphate-buffered saline; RA: rheumatoid arthritis; Tc: CD8^+^ cytotoxic T cell; Th: CD4^+^ helper T cell; TNFα: tumor necrosis factor alpha; WT: wild-type.

## Competing interests

The authors declare that they have no competing interests.

## Authors’ contributions

All authors made substantial contributions to the conception of the experiments and were involved in drafting the manuscript and revising it critically for important intellectual content. CE was involved in the design of the study and performed the AIA induction, histological examination, T-cell proliferation, flow cytometry, measurements of mBSA antibodies, and the statistical analyses. PC, AK, CO, and HC were involved in the design of the study. ML was involved in the design of the study and performed the ovariectomies, tissue collection, and statistical analyses. AB performed the breeding, genotyping, ovariectomies, tissue collections, and single-cell preparations. HF performed neutrophil assay, tissue collection, and single-cell preparation. AA, AS, and UI performed tissue collection, single-cell preparation, cellularity measurements, cytokine analysis, and flow cytometry. All authors read and approved the final manuscript.
